# Watching a Single Enzyme at Work Using Single-Molecule Surface-Enhanced Raman Scattering and DNA Origami-Based Plasmonic Antennas

**DOI:** 10.1021/acsnano.4c03384

**Published:** 2024-07-29

**Authors:** Yuya Kanehira, Sergio Kogikoski, Evgenii Titov, Kosti Tapio, Amr Mostafa, Ilko Bald

**Affiliations:** † Institute of Chemistry, University of Potsdam, 14476 Potsdam, Germany; ‡ Dynamics of Molecules and Clusters Department, J. Heyrovský Institute of Physical Chemistry of the CAS, Dolejškova 3, 18223 Prague, Czech Republic

**Keywords:** surface-enhanced Raman scattering, single molecules, DNA origami, enzymes, horseradish peroxidase

## Abstract

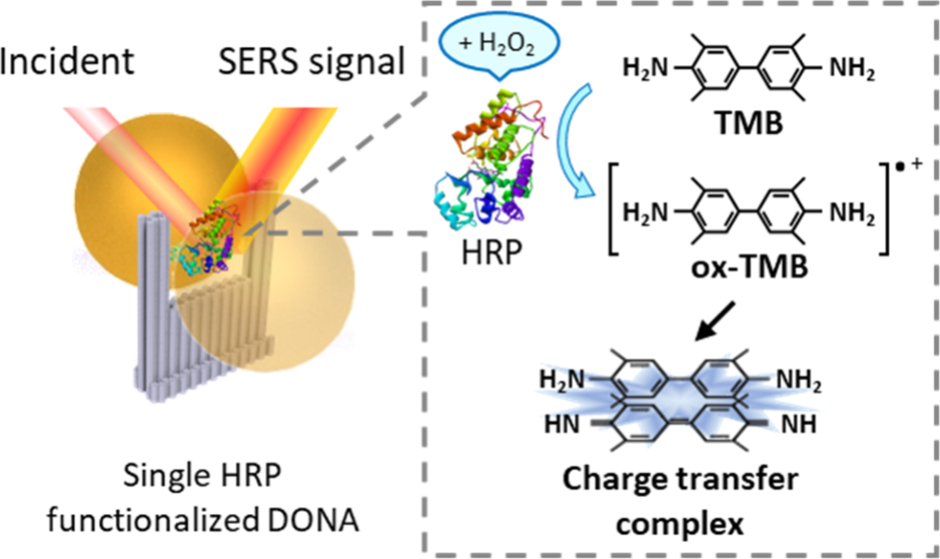

The detection of a single-enzyme catalytic reaction by surfaced-enhanced Raman scattering (SERS) is presented by utilizing DNA origami-based plasmonic antennas. A single horseradish peroxidase (HRP) was accommodated on a DNA origami nanofork plasmonic antenna (DONA) containing gold nanoparticles, enabling the tracing of single-molecule SERS signals during the peroxide reduction reaction. This allows monitoring of the structure of a single enzymatic catalytic center and products under suitable liquid conditions. Herein, we demonstrate the chemical changes of HRP and the appearance of tetramethylbenzidine (TMB), which works as a hydrogen donor before and after the catalytic reaction. The results show that the iron in HRP adopts Fe^4+^ and low spin states with the introduction of H_2_O_2_, indicating compound-I formation. Density functional theory (DFT) calculations were performed for later catalytic steps to rationalize the experimental Raman/SERS spectra. The presented data provide several possibilities for tracking single biomolecules in situ during a chemical reaction and further developing plasmon-enhanced biocatalysis.

## Introduction

DNA origami is a technique invented in 2006 to fabricate DNA-based nanostructures.^1^ These structures are constructed from a long scaffold strand folded into a specific shape with the aid of several short staple strands. Such a strategy allows for the formation of various nanostructures with numerous functionalizations that can be introduced by hybridizing and modifying individual nucleotides with high spatial resolution.^2^ This technology enables attaching nanoparticles (NPs), small molecules, and proteins. Therefore, a great deal of functionalized DNA origami nanostructures have been applied to the study of drug delivery,^3^ antibodies,^4^ enzyme cascade reactions,^5^ Förster resonance energy transfer (FRET),^6^ and localized surface plasmon resonance (LSPR)-based surface-enhanced Raman scattering (SERS) detection.^7^ Such versatility allows the engineering of lab-on-DNA origami platforms, on which different physical-chemical studies can be realized in the few to single-molecule regimes.^8^

SERS is the phenomenon that enhances the Raman signal from molecules near metal nanoparticle surfaces.^9,10^ The current state of the art in SERS allows for single-molecule detection (SMD)^11^ that enables a very low limit of detection in biosensing and expands the understanding of the fundamental mechanisms underlying biological phenomena at the single-molecule (SM) level.^12^ However, there are mainly two nanoengineering requirements to achieve SMD: (i) fabrication of a “hot spot” to provide high signal enhancement by precisely controlling the positions of NPs and (ii) “direct placement” of analyte molecules into the hot spot to obtain efficient enhancement. DNA origami meets those requirements and provides high-performance substrates based on its nanometer-scale functionalization scheme. Therefore, many DNA origami-based SERS substrates for SMD were fabricated in the past decade.^13−28^ In our previous study, DNA origami nanofork (NF) was used to fabricate the DNA origami nanofork plasmonic antenna (DONA),^13^ and SERS demonstrated its potential to directly measure single protein molecules such as cytochrome c and horseradish peroxidase (HRP). While single-molecule SERS has recently advanced for monitoring small molecules over time,^29^ the SMD of proteins by SERS has focused only on the detection of proteins.^13,17,27^ Only recently, single cytochrome C molecules could be monitored over an extended time scale using DONAs.^30^

HRP is a heme protein involved in oxidation processes.^31−37^ In the presence of peroxides (mainly hydrogen peroxide: H_2_O_2_) and substrate molecules, electrons are transferred to peroxides and substrate molecules are oxidized. This cyclic catalytic reaction can be utilized in various applications such as biosensors,^38^ wastewater remediation,^39^ and medical kits.^40^ Additionally, SM-SERS detection has a high potential to provide information about individual molecules, which is impossible to obtain from bulk and ensemble SERS measurements.^41^ Therefore, detecting redox or catalytic reactions at the SM level could provide information about the underlying mechanism. Single enzymes have already been studied extensively using single-molecule fluorescence spectroscopy, which has revealed, for example, activity distributions, dynamic disorder, and refined interpretations of Michaelis–Menten kinetics.^42−45^ However, the technique relies on the detection of fluorescence by chromophores. Single-molecule SERS promises a direct, label-free observation of single enzymes, which will significantly broaden the scope of systems that can be studied at the single-molecule level; nevertheless, several challenges must be overcome to fully monitor the reactions of single enzymes over time by SERS.

In our previous spectroscopic SM HRP study, SERS signals from a single HRP were detected using spherical gold nanoparticles (AuSph) with nonresonant excitation wavelength (λ = 633 nm) in air.^13^ HRP Raman signals, principally those related to the heme center, such as the ν_4_ and ν_10_ bands, well known as Fe oxidation markers,^31−34^ and the ν_2_ band, which refers to the Fe spin state,^32−34^ were observed at the single-molecule level using the DONAs. Most of the detected characteristic bands were broadened, and it was found that a single HRP tends to be in the Fe^3+^ state.

Here, we extend this study to detect a single HRP catalytic reaction in a liquid environment under real-time catalytic conditions, while previous studies focused on the mere detection of proteins, and this was mostly under dry conditions. SM-SERS spectra of HRP were recorded that are indicative of single steps of the catalytic cycle by adding H_2_O_2_ and TMB solutions and recording time series SM-SERS spectra indicating the catalytic capacity of a single HRP. We have divided the work into four parts: First, we show a single HRP’s functionalization to NF. Second, SM HRP SERS signals were detected under nonresonant conditions in liquid by snapshot measurements, supporting the reproducibility of SM HRP SERS detection. Third, the SM HRP catalytic reaction was traced by time series measurements. This part also endorses the reproducibility of single-molecule HRP SERS detection during the catalytic reaction. The last part demonstrates a strategy to develop SM protein detection, focusing on peptide bands.

## Results and Discussion

### Functionalization of the NanoFork with a Single HRP

**Figure 1:**
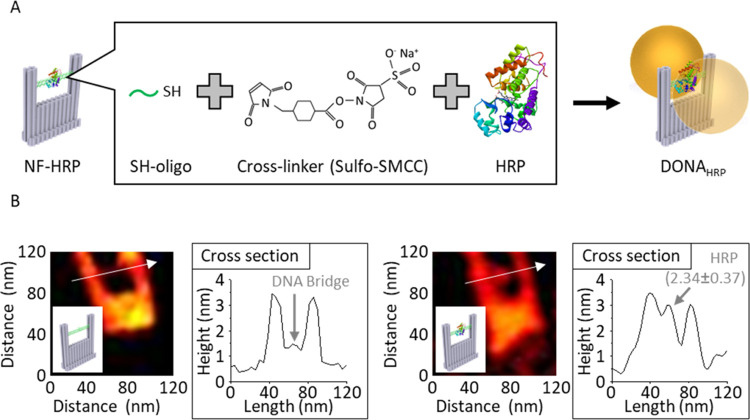
Overview of the functionalization of a single HRP. (A) Scheme of fabrication of NF-HRP and DONA_HRP_. (B) AFM image and cross section on the DNA bridge: (left) NF without HRP; (right) NF-HRP. The cross section indicated the height of HRP on NF to be 2.34 ± 0.37 nm.

As the first step, a single HRP was conjugated to NF. Herein, DNA origami is a platform for precisely placing molecules and NPs. The NF is used to sandwich a single HRP between two metallic NPs.^13^ Briefly, the fabrication of the SERS substrate possessing a single HRP (DONA_HRP_) was done in three steps: (a) folding of the NF without a staple strand at the specific location along the bridge, where the plasmonic hot spot will be generated, (b) functionalization of a single HRP to the empty strand location (the fabrication of NF-HRP) and (c) functionalization of NPs to NF-HRP resulting in DONA_HRP_. For the fabrication of NF-HRP, the conjugation of HRP to NF was performed by the sulfosuccinimidyl 4-(N-maleimidomethyl)cyclohexane-1-carboxylate (Sulfo-SMCC) cross-linker.^6,13^ HRP was covalently coupled to the NHS ester group of Sulfo-SMCC via its lysine residues, and simultaneously, the maleimide group of the cross-linker was bound to a thiolated staple strand, which in turn is incorporated into the DNA bridge of NF. At least one of the six lysines present in HRP is expected to be functionalized. Figure 1 illustrates the fabrication scheme of NF-HRP and DONA_HRP_, as well as the corresponding atomic force microscopy (AFM) images of NF-HRP. For example, Figure 1B (right) shows a single HRP on the DNA bridge between the arms of NF with a height of 2.34 ± 0.37 nm. A larger AFM image showing several functionalized DONA_HRP_s is given in the Supporting Information (SI), Figure S1. The functionalized HRP and the DONA_HRP_ catalytic activity were tested via an assay where the enzyme was mixed with tetramethylbenzidine (TMB) and H_2_O_2_. When the enzyme is active, the solution turns rapidly blue, showing the presence of oxidized TMB. The procedure and photographs of the obtained results showing that the enzyme was active are given in the SI.

### Single HRP Snapshot Measurement

**Figure 2:**
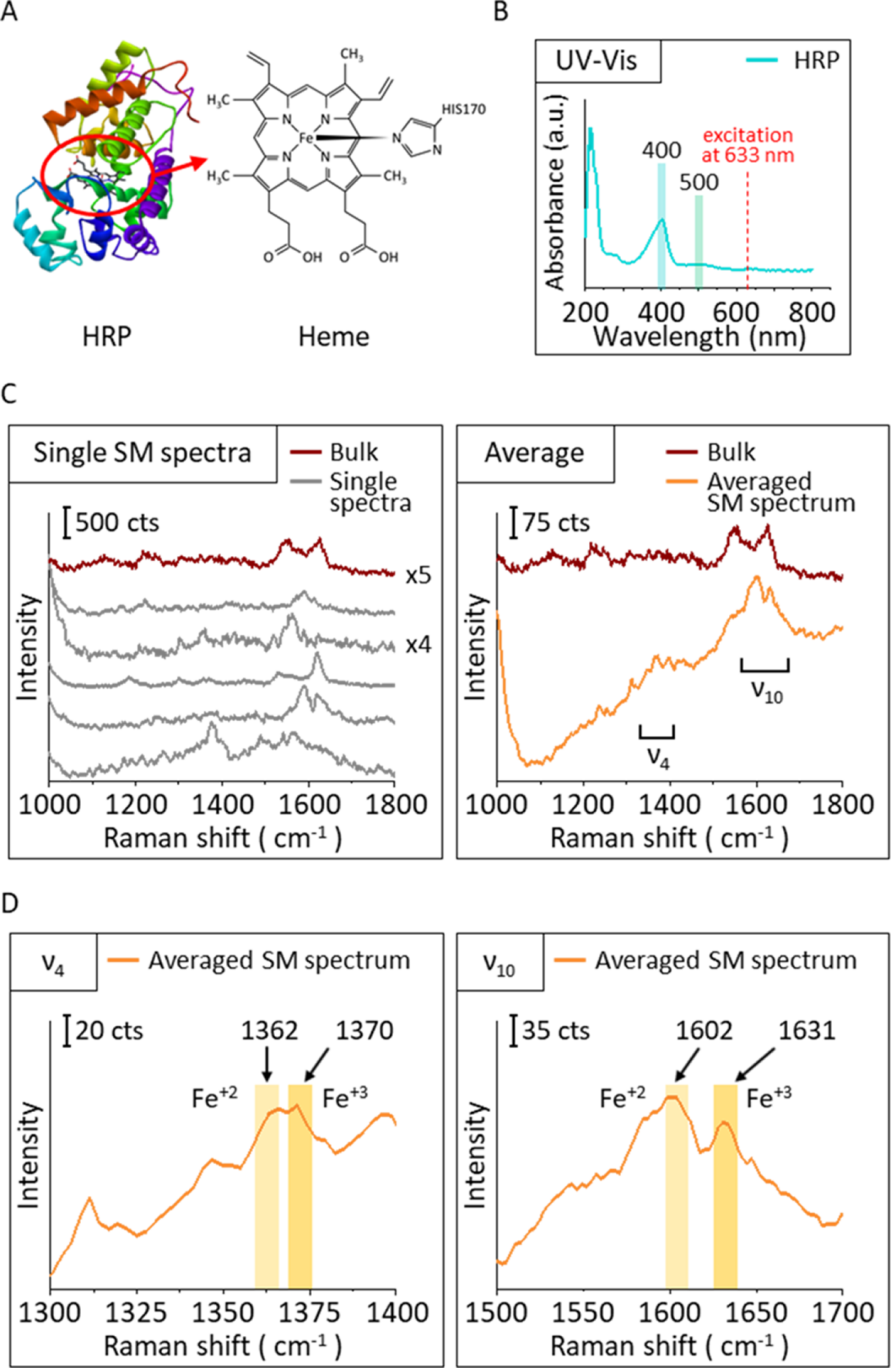
Spectroscopic properties of HRP. (A) Structure of HRP and heme unit in HRP. The HRP model was adapted from [10.2210/pdb 1HCH/pdb]. (B) UV–vis absorption spectrum of HRP. (C) Single-molecule SERS signals of HRP: (left) single SM spectra; (right) averaged SM spectrum; top curves show the bulk reference spectrum. (D) Averaged SM-SERS spectra of HRP, zoomed into ν_4_ and ν_10_ bands.

In the second step, a single HRP SERS signal is detected in liquid conditions by sandwiching the HRP between two NPs, thus forming a DONA structure. In our previous study, we fabricated several DONAs showing the SERS active range of 488–633 nm for SMD.^14^ The present study used AuSph DONA to detect a single HRP SERS signal. Figure 2A shows the structure of HRP and the heme unit in HRP, and Figure 2B shows the UV–vis absorption spectrum. HRP has a strong absorption band at 400 nm, the Soret band or B band, originating from the π–π* transition of porphyrin, and a set of weak bands around 500 nm, which are the Q bands, due to the electronic transition between the electronic ground state and first electronic excited state.^46^ The Q-band can be used for resonant Raman scattering in SMD by employing a spherical silver nanoparticle (AgSph) DONA with the LSPR at 488 nm and a suitable laser tuned to the same wavelength.^14^ However, AgNPs are reported to have catalytic abilities to oxidize hydrogen donor substrates;^47^ therefore, AuSph DONA with the LSPR at 633 nm was utilized here.

Figure 2C shows single SM-SERS spectra of HRP (“Single spectra” in the figure) and averaged single-molecule SERS spectrum of HRP (“Averaged SM spectrum” in the figure) in the liquid condition, compared to a bulk HRP Raman spectrum recorded in the air (“Bulk spectrum” in the figure). Unfortunately, HRP Raman spectra in solution are challenging to record and yield only weak or broadened signals. Thus, a comparison is made with the bulk spectrum obtained from HRP crystals. Regardless, the peaks to be observed are expected in the same spectral region as was confirmed by comparison of DONA_HRP_ measurements under dry and liquid conditions. The assignments of HRP signals^31−34^ are shown in Table S1 in the Supporting Information (SI). Snapshot measurements were performed by the correlation of Raman mapping and scanning electron microscopy (SEM) measurements, and 10 DONAs were measured for each substrate. The correlation image is shown in Figure S1. A more detailed description of how the correlation is performed can be found elsewhere.^7,24^ At SMD, peak fluctuations have been reported to give rise to broadened bands in the bulk spectrum.^48^ Thus, the obtained spectra showing signals at the same positions as the bulk spectrum were considered to be SM spectra from the analyte. Further results will only show the average of all observed SM-SERS events so that the spectra’s differences are more easily identified. Nevertheless, some SM spectra are given in the SI.

In the bulk spectrum, Figure 2C, most characteristic bands are from the porphyrin ring of the heme unit, and peptide bands are not observed because the intense bands of the porphyrin ring obscure them.^32^ While the detection of the ν_4_ band around 1370 cm^–1^, which is known to be an oxidation state marker^31−34^ could not be obtained in bulk, other bands around 1550–1640 cm^–1^ (ν_2_, ν_10_), which are known as the spin state marker (ν_2_)^32−34^ and oxidation state marker (ν_10_),^31−34^ were observed. The weak appearance of the ν_4_ band can be due to the resonant Raman scattering contribution of the porphyrin ring.^49,50^

In contrast to bulk spectra, single HRP SM-SERS spectra showed a lot of peak fluctuations, such as the blinking of bands, as shown in Figure 2C (left). For example, the signal from a single SM spectrum showed the ν_30_ band around 1165 cm^–1^, which is assigned to the porphyrin ring,^32^ Other spectra showed bands around 1275 and 1505 cm^–1^ that are not from the porphyrin ring.^31,51^ Additionally, the ν_2_ band at 1550–1575 cm^–1^ and the ν_10_ band at 1605–1640 cm^–1^, clearly observed in the bulk spectrum, were weakly detected in some spectra. The most interesting observation was detecting the ν_4_ band at around 1375 cm^–1^. This band was not obtained in the bulk spectrum, but Fe^2+^ (at 1359 cm^–1^) and Fe^3+^ (at 1375 cm^–1^) states^31−34^ were obtained in several SM-SERS spectra. Focusing on the averaged SM spectrum, single HRP signals show both Fe^2+^ and Fe^3+^ states (ν_4_ band showed 1362 and 1370 cm^–1^, and ν_10_ band showed 1602 and 1631 cm^–1^). Additionally, spin state marker bands around 1550–1575 cm^–1^ often show many peak fluctuations.

According to previous reports, heme groups are highly sensitive to both oxidation and spin state to changes in the external environment, such as the presence of chemical reagents,^15,33,52,53^ temperature variation,^54^ pH change,^55^ applied pressure,^56^ conjugation with RNA/DNA,^57^ and the interaction with the surface due to the molecular adsorption to the metal.^36^ During sample preparation, the target molecule is exposed to different and complex environments, such as different buffers and DNA-coated NPs, which could lead to modifications of the iron redox state from +3 to +2. Additionally, possible plasmonic-induced products, such as amorphous carbon, were reduced by decreasing the excitation laser powers to a minimum to observe signals.^58^ This can lead to spectral changes compared to typical Raman spectra such as the absence of ν_2_ or ν_10_ bands in some single SM spectra and the detection of several iron states. We could not determine the most significant factor contributing to the peak fluctuations here. Still, detecting characteristic heme protein signals indicates that HRP spectra were obtained at a single molecule level in liquid conditions.

### Single HRP Time Series Measurement during Catalytic Reaction

**Figure 3:**
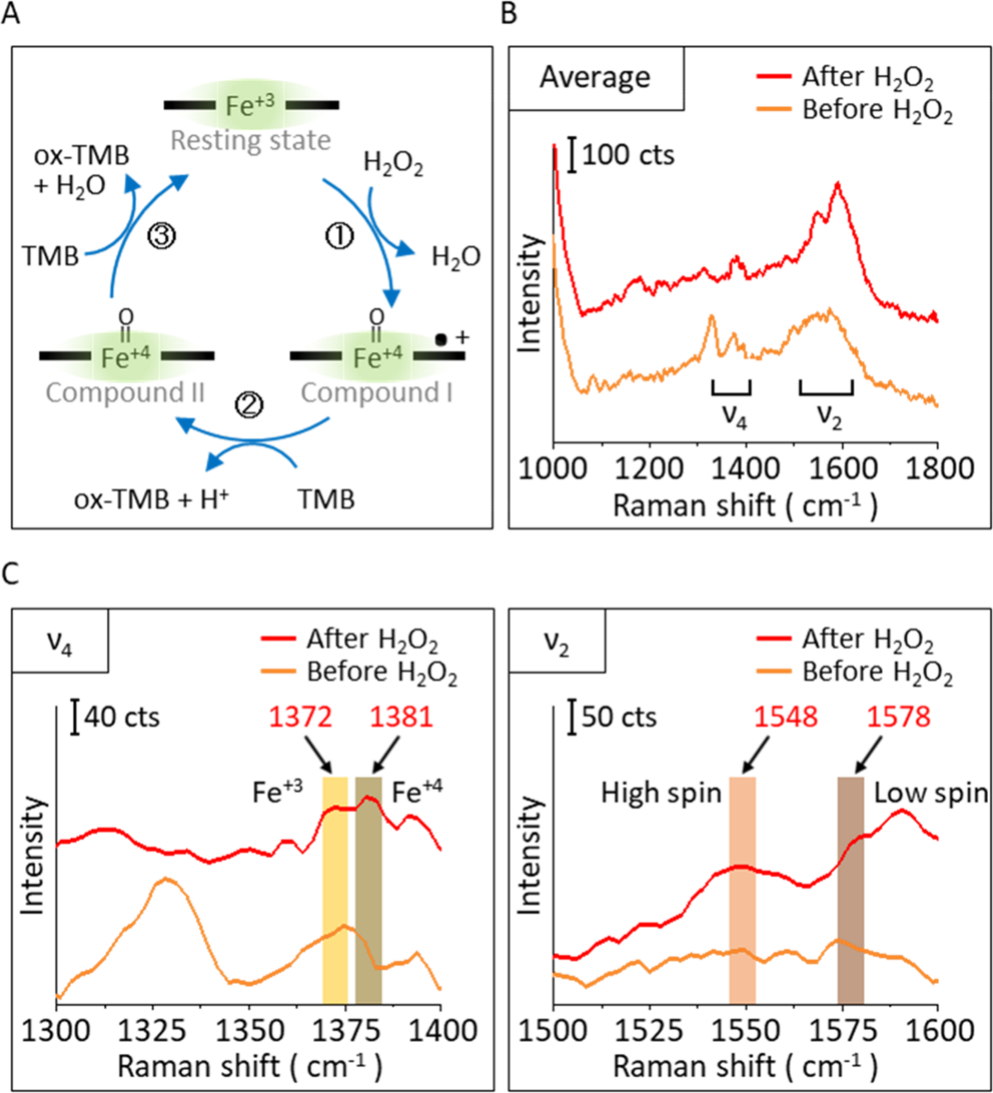
Single HRP spectroscopic properties during H_2_O_2_ reduction. (A) Scheme of the catalytic reaction of HRP. (B) Averaged single-molecule SERS signals of HRP during catalytic reaction by H_2_O_2_ solution. Spectra are shown before (orange) and after (red) the addition of an H_2_O_2_ solution. (C) Averaged SM-SERS spectra of HRP, zoomed into ν_4_ and ν_2_ bands.

In the third step, a single HRP catalytic reaction was traced using H_2_O_2_ and 3,3′,5,5′-tetramethylbenzidine (TMB). Figure 3A shows a schematic image of the catalytic reaction of HRP. TMB is a chromogenic substrate used for chemical staining to visualize peroxidase enzymes, such as in enzyme-linked immunosorbent assay (ELISA).^59,60^ When the peroxidase enzyme, such as HRP, acts in solution, TMB works as a hydrogen donor and reducing agent, converting Fe^4+^ to Fe^3+^, simultaneously turning blue (oxidation of TMB and formation of a charge transfer complex). The detection of this blue analyte enables the determination of the concentration of a target analyte (via HRP) since the intensity of the TMB signal is proportional to the amount of the analyte.^59,60^ We used the same test to prove that the enzyme is active after incorporation into the DNA origami nanofork (SI).

To detect the catalytic reaction, H_2_O_2_ was first added to single HRP-functionalized DONAs, and SERS signals were collected before and after adding H_2_O_2_ solution during time series measurements. Time series measurements were performed using the correlation between AFM and Raman measurement,^7,24^ and 10 DONAs were measured. The correlation image is shown in Figure S2. As in the case of the snapshot measurement, the obtained time series spectra that showed signals at the same positions as the bulk spectrum were considered to be SM spectra from the analyte. Figure 3B shows the averaged single-molecule SERS spectra of HRP before and after addition of the H_2_O_2_ solution. The time trace 3D map of one of the analyzed dimers is given in Figure S3. There, we observe the signal before and after adding H_2_O_2_, when the signal strongly fluctuates due to the manual addition of the H_2_O_2_ solution. From the maps, the SM-SERS spectra from each time a signal is generated are extracted, both from before and after the addition and from all of the extracted signals. Also, in Figure S3, the average signal collected from individual DONA_HRP_s is given for the cases before and after the addition of H_2_O_2_ addition.

In the spectrum before adding the H_2_O_2_ solution, HRP signals were obtained, including the band at 1330 cm^–1^, which is not from the porphyrin ring,^31^ ν_4_ band at 1374 cm^–1^, ν_20, 29_ band at 1395 cm^–1^,^32^ and broadened ν_2_ bands around 1550–1575 cm^–1^. The ν_10_ band was obtained at 1630 cm^–1^ in a single SM spectrum, as shown in Figure S3, but it was not observed in the averaged SM spectrum. Peak fluctuations were typically observed during time series measurements compared to snapshot spectra (Figure 2C), indicating that those signals are from a single HRP.^61,62^

After adding the H_2_O_2_ solution, HRP signals were also obtained, such as the band ν_30_ around 1165 cm^–1^, bands around 1230 and 1268 cm^–1^, that are not from the porphyrin ring,^31^ ν_4_ bands, ν_20, 29_ band, and ν_2_ bands. The ν_10_ bands were detected around 1630 cm^–1^ in several single SM spectra (Figure S3) but were also not observed in the averaged SM spectrum due to their low probability of appearance. Focusing on the ν_4_ bands, the band at 1372 cm^–1^, indicating the Fe^3+^ state, and the band at 1381 cm^–1^, indicating the Fe^4+^ state, were observed. In contrast, only the band at 1374 cm^–1^ (indicating the Fe^3+^ state) was obtained in the spectrum before adding H_2_O_2_ solution. Additionally, while two ν_2_ bands, indicating the existence of high and low spin states, were obtained both before and after adding H_2_O_2_ solution, the band at 1578 cm^–1^, indicating a low spin state, was slightly higher in the spectrum after adding H_2_O_2_ solution. HRP is reported to form compound I when it reacts with H_2_O_2_, oxidizing the Fe^3+^ to Fe^4+^ and changing to a low spin state.^33−35,52−54^ Therefore, those observations indicate the successful detection of the first step of the HRP catalytic reaction (oxidation of HRP in Figure 3A).

**Figure 4:**
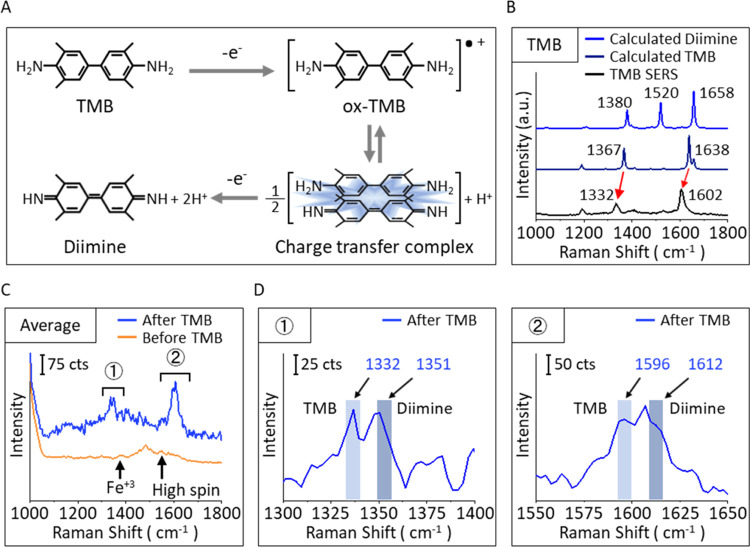
Single HRP spectroscopic properties during the complete H_2_O_2_ reduction cycle. (A) Scheme of oxidation of TMB. (B) TMB calculated spectra and SERS spectrum. (C) Averaged SERS signals during cyclic catalytic reaction by a TMB solution. Spectra are shown before (orange) and after (blue) adding the TMB solution. (D) Averaged SERS spectra after adding TMB solution, zoomed into two characteristic TMB bands.

As the next step in detecting the cyclic catalytic reaction of HRP, a TMB solution containing both TMB and H_2_O_2_ (see the Methods section) was added to single HRP-functionalized DONAs. As in the study of adding H_2_O_2_ solution, in previous paragraphs, SERS signals were collected before and after adding the TMB solution during the time series measurements. The number of measured DONAs was also 10. Figure 4A shows a schematic image for the oxidation of TMB, 4-B shows the TMB SERS spectrum, compared with calculated spectra of TMB (diamine) and diimine, which form the charge transfer complex, and Figure S4 shows characteristic TMB and diimine vibrational modes. In the TMB SERS spectrum, three characteristic bands were observed at 1191, 1332, and 1602 cm^–1^, attributed to the ring C–H bending mode, inter-ring C–C stretching mode,^47,55^ and a combination of ring C–C stretching,^47,55^ C–H bending,^47,55^ and N–H bending modes, respectively. Those characteristic bands were also obtained in the calculated TMB spectrum. Compared to the calculated TMB, several peak shifts and new bands were observed in the diimine spectrum, such as blue-shifting of the inter-ring C–C stretching mode (1367–1380 cm^–1^), a combination of ring C–C stretching, C–H bending and N–H bending modes (1638–1658 cm^–1^) and a new band at 1520 cm^–1^ which can be attributed to the combination of ring breathing, inter-ring C–C stretching and C–H bending modes, indicating structural changes upon oxidation.

Our initial assessment was that adding TMB to the mixture would reduce the Fe^4+^ to Fe^3+^ and that we could now observe the end of the catalytical cycle. However, we have not predicted that TMB’s contact with the DONA_HRP_ would provide a strong SERS signal. Principally, because TMB was not observed in the system that did not contain the HRP, Figure S6 was one reason for choosing an AuSph DONA. Nevertheless, the TMB, the charge transfer complex, and the diimine provided intense SERS signals that obscured the protein signals.^55^ Thus, we aimed to detect those TMB signals, principally the diamine, to observe the cyclic catalytic reaction of HRP, focusing on those bands that are two blue-shifting and new bands.

Figure 4C shows the averaged single-molecule SERS spectra of HRP before and after the addition of the TMB solution, and each single SM spectrum is shown in Figure S5. Typical single HRP signals were obtained in the spectrum before adding the TMB solution. After the TMB solution was added, only TMB signals were obtained, and HRP signals were not detected (Figure S5). A control experiment was carried out to check the interaction between AuSphs and TMB by adding a TMB solution to the DONA to check the interaction between AuSphs and TMB. Figure S6 shows the SERS signal from non-HRP-functionalized DONA after the addition of the TMB solution. No meaningful signals were detected; therefore, the signals shown in Figure 4 indicate detection of the catalytic reaction of HRP at the SM level. In the zoomed average spectrum after adding the TMB solution in Figure 4D, the broadened bands at 1332–1351 and 1596–1612 cm^–1^ are shown (the new band for 1520 cm^–1^ in the calculated diimine spectrum was not observed).

According to the assignment described above,^47,55^ this result indicates that TMB and diimine signals were obtained from a single HRP-functionalized DONA after adding TMB solution. The catalytic capacity of a single HRP can explain this. A single HRP can catalyze only a limited amount of H_2_O_2_ and TMB. Thus, two characteristic bands indicating the presence of TMB and diimine were obtained. However, the control experiment (Figure S6), in which the TMB solution was added to non-HRP-functionalized DONAs, showed no TMB signals (the same TMB solution was used in both experiments). This can be associated with the Raman activity of TMB molecules under different environments. Perumal et al. reported the TMB oxidation process at different pH conditions.^55^ They showed the pH dependency of TMB SERS signal intensities, demonstrating that TMB SERS signals were significantly enhanced under low-pH conditions. In contrast, no signals were obtained under high-pH conditions. Here, to examine the pH dependency of TMB SERS signals, TMB SERS signals were detected by time series measurement from AuNPs aggregation with the addition of HCl to reduce the pH value. Figure S7 shows TMB SERS signals under different pH conditions. The low-pH condition provided a higher TMB signal intensity. Based on those results, we assume that the catalytic reaction of a single HRP induces the reduction of pH value because of H^+^ release during the oxidation of TMB in the hot spot, improving both the Raman activities of TMB and diimine. Due to the catalytic capacity of a single HRP, diimine and nonoxidized TMB can exist in the hot spot, and both signals were enhanced. Unfortunately, we cannot investigate the pH value in the hot spot. However, the observation of strong TMB bands indicates that the local pH could possibly change during the reaction.

Lastly, apart from a catalytic reaction of a single HRP, we also assigned the SERS bands not originating from the porphyrin ring in both snapshot and time series measurements. According to the assignment of proteins, the bands around 1238–1275 cm^–1^ are attributed to amide III bands.^31^ Those bands are assigned to the C–N stretching vibration coupled to N–H bending of the peptide bonds. Therefore, they are sensitive to the secondary structure.^51,63^ Garcia-Leis et al. reported the SERS detection of Amyloid β-peptide and showed the plasmonic interaction for amide III bands.^64^ According to their investigation, peptides can form/reform the secondary structure for both the α-helix and β-sheet when adsorbing to metal NPs. The obtained spectra in our study also showed both signals at 1251 cm^–1^ for α-helix and 1235–1238 cm^–1^ for β-sheet structures.^51,64^ Thus, this observation indicates the possibility of investigating the protein behavior under the plasmonic environment at the SM level. Moreover, our measurements enable monitoring in a liquid environment, which allows us to study the properties of biomolecules in the native environment compared to dry conditions, which typically results in thermal denaturation or degradation.^65^ To realize those investigations, Figure S8 shows the resonant contributions of peptides.

The normal Raman spectra of HRP in Figure S8 show the excitation wavelength dependency, which indicates that 488 nm excitation is suitable to detect porphyrin ring bands, whereas 785 nm is ideal for detection of peptide bands such as phenylalanine or amide III.^31^ The detection of peptide SERS signals under 488 and 633 nm excitation at the SM level can be related to the placement and conformation of HRP within the hot spot between NPs. At the bulk detection level, especially under 488 nm excitation, peptide bands were not obtained due to the intense porphyrin rings bands,^32^ but SM detection is enabled by the picocavities that involve direct metal-to-molecule interaction,^29^ which will allow the detection of bands that are otherwise unable to be detected in bulk and standard SERS methods. In this study, we did not use 785 nm excitation due to the lack of plasmonic resonance of the DONAs at this excitation.^14^ However, the utilization of NPs with sufficient enhancement factor at 785 nm excitation, such as bigger sized AuNPs,^66^ rod NPs,^27^ or star NPs possessing sharper tips,^67^ can be used in future studies to investigate those peptide properties at the SM level.

## Conclusions

This work aimed to detect the HRP catalytic reaction at the SM level, where a single HRP molecule was conjugated to NF. AFM images demonstrated the successful conjugation of a single HRP to the DNA bridge of the NF, showing a height of 2.34 ± 0.37 nm. Second, several NPs were functionalized to NF-HRP to fabricate DONA_HRP_, and HRP SM-SERS signals were detected under nonresonant conditions in an aqueous buffer. SM-SERS spectra showed characteristic HRP signals, indicating the reproducibility of SM HRP SERS detection. Third, the catalytic reaction of a single HRP was traced by using the H_2_O_2_ solution and TMB solution. SM-SERS spectra after adding the H_2_O_2_ solution showed signals of compound I, indicating the oxidation of HRP. After adding the TMB solution, SM-SERS spectra showed both TMB and diimine signals, indicating the presence of TMB and diimine at the SM catalysis level. This is ascribed to the catalytic capacity of a single HRP and Raman activities of chromogenic substrates induced by the reduction of pH in the hot spot. The results show that DNA origami is versatile in elucidating enzymatic cycles; herein, we could individually trace two of the three catalytical steps of HRP, and further analysis of individual spectra will reveal individual enzyme variability. Here, we observed that the average signals show a mixed system containing the heme center in oxidized and reduced states, possibly because of the averaging process. An individualized analysis will enhance comprehension of what makes the enzyme active.

Lastly, a strategy to investigate the peptide structure at the SM level was discussed. The excitation wavelength dependency for complex biological molecules indicated a high potential to detect peptide SM-SERS signals. The versatility of DNA origami structures concerning the functionalization with various materials, utilization for measurements under different conditions, and detectability of diverse components provides many possibilities for further developing a wide range of interdisciplinarity research fields. Nevertheless, the DONA system is still under development and in the process of optimization. Problems such as observing the TMB signal instead of the HRP should be overcome by other means, such as coupling single-molecule spectroscopy with electrochemical controls, avoiding the need for chemical agent addition, which, in our case, compromised the full observation of the Fe cycle. As the next step, we plan to study the variability of the enzymatic activity at the single-molecule level. For this, computational tools that can analyze a large number of spectra and possibly study the time evolution of the spectral changes are needed. Thus, a profound comprehension of enzyme changes and possible links to degenerative processes will be possible.

## Methods

### Sample Preparation

#### NanoFork Folding and HRP Functionalization

The protocol for the fabrication of NF followed our previous study.^13^ Briefly, to fabricate the NF, possessing a single HRP, first, the folding solution was prepared by 67.5 μL of Milli-Q water, 19 μL of 100 μM staple solution, which is missing a staple strand at the specific location of the hot spot (s161), 10 μL of buffer (10xTAE/150 mM MgCl_2_), and 2.5 μL of scaffold solution (100 nM m13mp18). A thermocycler heated the folding solution to 80 °C and gradually cooled it to 20 °C over 12 h. The purification process to remove the excess and nonhybridized oligos was performed by centrifuging the folding solution and four times excess Milli-Q water with a 100 kDa filter (5000 rpm, 5 min). Afterward, the filtered water was removed, and additional Milli-Q water was added. The purification was repeated three times, and the remaining solution in the filter was finally recovered.

Second, to functionalize a single HRP to NF, a thiolated single strand (s161) and SMCC linker were used. To conjugate a single HPR to a thiolated s161 single strand, 4 μL of 100 μM thiolated s161s single strand was mixed with 1 μL of 100 mM tris(2-carboxyethyl)phosphine (TCEP) and incubated for 10 min at room temperature to cleave the disulfide bond. Afterward, the cleaved s161 strand solution (5 μL) was mixed with 1 mg of Sulfo-SMCC and 40 μL of 10 μM HRP in 50 mM phosphate buffer solution (PBS). The mixture was stirred in a vortex and incubated for 24 h in a refrigerator (4 °C). The purification to remove excess s161 strands was performed by centrifugation with 10 kDa centrifugal filters. The incubated mixture and four times excess Milli-Q water were added to the filter tube and centrifuged (13,000 rpm, 10 min). The filtered water was removed, and additional Milli-Q water was added again. The purification was repeated two times, and the remaining solution in the filter, the single HRP-functionalized s161 strand solution, was finally recovered.

Lastly, to fabricate NF-HRP, both NF which is missing an s161 strand solution and HRP-functionalized s161 strand solution were mixed. The concentration ratio between NF and HRP was 1:100. A thermocycler heated the mixed solution to 37 °C and gradually cooled it to 20 °C for 3 h. The purification to remove excess HRP was performed by centrifugation with a 100 kDa centrifugal filter. The mixture solution and four times excess Milli-Q water were added to the filter tube and centrifuged (5000 rpm, 5 min). The filtered water was removed, and additional Milli-Q water was added again. The purification was repeated twice, and the remaining solution in the filter, the NF-HRP solution, was finally recovered. DNA sequence of s161 is shown in Table S2.

#### DNA Coating

Coating AuNPs with DNA was following our previous study.^14^ To perform the coating, two coating strand sequences were used, i.e., a 5′-SH-T_28_-3′ sequence (TTT) and a 5′-(GTT)_8_T_4_-SH-3′ sequence (GTT). First, the volume and concentration of the AuNPs solution were adjusted with Milli-Q water (26.5 μL and 0.25 nM). Second, to cleave the disulfide bond in the coating strands, 4 μL of 100 μM coating strand was mixed with 1 μL of 100 mM TCEP and incubated at room temperature for 10 min. Afterward, the coating solution (26.5 μL), the cleaved coating strand solution (5 μL), and 3.5 μL of 0.2% sodium dodecyl sulfate (SDS) were mixed and incubated for two h in the freezer (temperature below −18 °C). The coating process was applied to both of the coating strands. The purification process to remove the excess coating strand was performed by centrifuging the coating solution with 10 times excess Milli-Q water (3000 rpm, 5 min). After centrifugation, the supernatant was removed, and additional Milli-Q water was added. The purification was repeated twice, and finally, the DNA-functionalized AuNPs solution was extracted.

#### Fabrication of DONA

The protocol for the fabrication of DONA was following our previous study.^13^ Briefly, DONAs were fabricated by hybridizing the coating strands on AuNPs and the complementary sequence on NF. The DNA-functionalized AuNPs and NF-HRP solutions were mixed and diluted in a 5 mM MgCl_2_ solution. The concentration ratio between AuNPs and NF was 1.5:1. A thermocycler heated The mixture to 40 °C and gradually cooled it to 20 °C over 3.5 h. The purification process to extract the dimer structures was carried out by agarose gel electrophoresis. The DONA solution was run in 1% (w/v) agarose gel with 5 mM MgCl_2_ over 60 min at 80 V. The dimer band in the gel was cut out and squeezed between glass plates wrapped in parafilm. The resulting solution was collected and used for the next set of experiments.

#### Deposition on Silicon

The DONA solution was deposited on a silicon chip with a scratch as a positional marker to characterize the DONA structure in atomic force, Raman, and scanning electron microscopy (SEM). A diamond cutter drew the scratch. After drawing, the silicon chip was cleaned by a cotton stick in acetone and sonicated in ethanol for 30 s. The cleaned chip was plasma-treated for 10 min. The concentration of MgCl2 in the DONA solution was adjusted to 50 mM for the deposition. DONA solution was deposited on a plasma-treated silicon chip and incubated for 1 h. The DONA-covered silicon chip was washed with an ethanol–water solution (*V*_EtOH_/*V*_H2O_ = 1:1) and dried by blowing with an air gun.

### Measurements

#### AFM

A Bruker Multimode 8 atomic force microscope (Billerica, Massachusetts) was used for AFM measurements. DONAs were deposited on the silicon chip and measured by the ScanAsyst Tapping mode with a 70 kHz resonant frequency tip. The scan area was 20 μm with a 0.3 Hz scan rate.

#### Raman Spectroscopy

A Witec α 300 Raman microscope (Witec, Tlm, Germany) was used for all Raman measurements. A 100x objective (Olympus MPlanFL N) was used for the bulk measurement in air. Excitation lasers were at λ = 488, 532, 633, and 785 nm, and laser powers were 200 μW, 300 μW, 2 mW, and 10 mW, respectively. Integration time was 4 s. A 63x immersion objective (ZEISS) was used for the SM-SERS measurement in a liquid environment. Excitation lasers were at λ = 633 nm, and laser powers were at 2.5 mW. Integration time was 4 s for snapshot measurement and 1 s for time series measurement. The irradiation time for the time series measurement was 300 s. The bulk sample was simply measured on top of nonmodified HRP crystals deposited over a Si chip.

Regarding the sample preparation for SM-SERS measurement in liquid condition, the sample (DONAs on Si chip) was immobilized in the glass chamber by glue (CHIPQUIK, ED10C-20G) and incubated for 2 h. After the incubation, 3 mL of 50 mM PBS was added, and the sample was completely immersed in the buffer.

Two reaction initiators were used to detect the single HRP catalytic reaction. In both studies, 1 mL of the reaction initiator (H_2_O_2_ or TMB solution) was added during the measurement. The H_2_O_2_ solution was fabricated by mixing PBS and H_2_O_2_. A 9.5 μL portion of 30% H_2_O_2_ was added to 20 mL of 50 mM PBS. TMB solution was fabricated by mixing PBS, dimethyl sulfoxide (DMSO), TMB, and H_2_O_2_. First, TMB was dissolved in DMSO (40 mM TMB). Second, 250 μL of 40 mM TMB in DMSO solution was added to 20 mL of 50 mM PBS. Lastly, 9.5 μL of 30% H_2_O_2_ was added to the mixture. The concentration of TMB in the “TMB solution” was 0.5 mM, and the percentage of the amount of H_2_O_2_ was 1.56 × 10^–4^%.

#### SEM Measurement

A scanning electron microscope (ZEISS UltraPlus) was used for the SEM measurement. The measurement was carried out under high-vacuum conditions. The accelerating voltage and spot size were set to 30 kV and 2.0 nm, respectively.

#### Simulation

TMB diamine and diimine neutral forms were optimized in the electronic ground state using the B3LYP functional^68,69^ augmented with the D3(BJ) dispersion correction^70^ and the 6-311++G** basis set.^71^ In addition, (implicit) solvation in water was assumed and described with the polarizable continuum model (PCM).^72,73^ No imaginary frequencies were found for the optimized geometries, confirming the minimum nature of the located structures. The calculations were performed using Gaussian 16.^74^ Nonresonant Raman stick spectra were computed using the Freq(Raman) keyword and further broadened with Lorentzians as
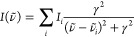
Here, *I*_*i*_ and ν̃_*i*_ are Raman activity and wavenumber of mode *i*, respectively (calculated with Gaussian 16), and γ is a broadening parameter (chosen to be 4 cm^–1^ in this work). The computed vibrational wavenumbers were not scaled.

## Supporting Information Available

Assignment of the most characteristic SERS bands in HRP (Table S1); large-scale AFM image and Raman SEM correlation image (Figure S1); overview of AFM and Raman correlation image for time series measurement (Figure S2); scheme of the single-molecule SERS data analysis (Figure S3); simulation of theoretical TMB and diimine vibrational modes (Figure S4); single-molecule SERS signals of HRP during the cyclic catalytic reaction (Figure S5); control experiment to investigate the interaction of AuNPs with TMB (Figure S6); pH dependency of TMB SERS signal (Figure S7); normal Raman spectra of bulk HRP at different excitation wavelength (Figure S8); DNA sequences of staple strands which were utilized for the functionalization of HRP (Table S2); and activity test for modified HRP (Figure S9) (PDF)
