# Supplementary material for: Watching a Single Enzyme at Work Using Single-Molecule Surface-Enhanced Raman Scattering and DNA Origami-Based Plasmonic Antennas
Source: ACS Nano. 2024 Jul 29;18(31):20191–200. doi: 10.1021/acsnano.4c03384 (PMC11308918; doi:10.1021/acsnano.4c03384)
Supplement: Supplementary file 1 — nn4c03384_si_001.pdf [file nn4c03384_si_001.pdf]

Watching a single enzyme at work using single-molecule surface-enhanced Raman scattering and DNA origami-based plasmonic antennas

Yuya Kanehira,[1] Sergio Kogikoski, [1] Evgenii Titov, [1] Kosti. Tapio,[1,#] Amr. Mostafa,[1] Ilko Bald[1,*]

[1]*Institute of Chemistry, University of Potsdam, 14476 Potsdam, Germany*

[2]*Dynamics of Molecules and Clusters Department, J. Heyrovský Institute of Physical Chemistry of the CAS, Dolejškova 3, Prague, 18223, Czech Republic*

[#]*Present address: Department of Neuroscience and Biomedical Engineering, University of Aalto, Espoo 02150, Finland*

*correspondence: bald@uni-potsdam.de

Table S-1. Assignment of the most characteristic SERS bands in HRP.[1–4]

Wavenumber (cm^{-1})	Assignments
1130	ν_{22} (Pyrrole half-ring)$_{sym}$$^{(1)}$
1165	ν_{30} (Pyrrole half-ring)$_{sym}$$^{(1)}$
1358-1385	ν_{4} (Pyrrole half-ring) $^{(1), (2), (3)}$
	*oxidation marker: 1358 cm^{-1} (Fe^{+2}), 1375 cm^{-1} (Fe^{+3}), 1382 cm^{-1} (Fe^{+4})
1395	ν_{20} ,ν_{29} (Pyrrole quarter-ring)$_{sym}$$^{(1)}$
1478	ν_{3} (C_{α}-C_{m})$_{sym}$ from porphyrin ring$^{(1)}$
1550-1575	ν_{2}, ν_{11} (C_{β}-C_{β})$_{asym}$ from porphyrin ring$^{(1), (2), (3)}$
	*spin state marker: 1550 cm^{-1} (High spin), 1575 cm^{-1} (Low spin)
1605-1640	ν_{10} (C_{α}-C_{m})$_{asym}$ from porphyrin ring$^{(1), (2), (3)}$
	*oxidation marker: 1605 cm^{-1} (Fe^{+2}), 1630 cm^{-1} (Fe^{+3}), 1641 cm^{-1} (Fe^{+4})

ν: Stretching

$^{(1)}$: SERS spectrum, $^{(2), (3)}$:Raman spectrum

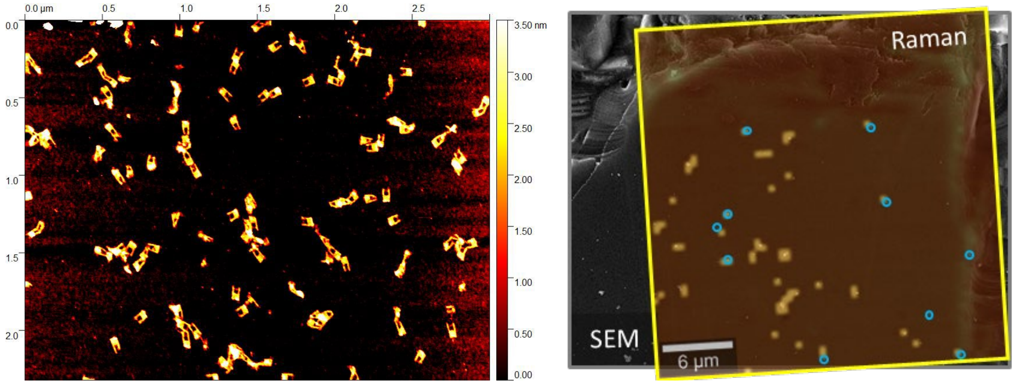

Figure S-1. Large scale AFM image showing several DNA origami nanoforks modified with HRP (NF_{HRP}). Overview of Raman imaging and SEM correlation image for snapshot measurement.

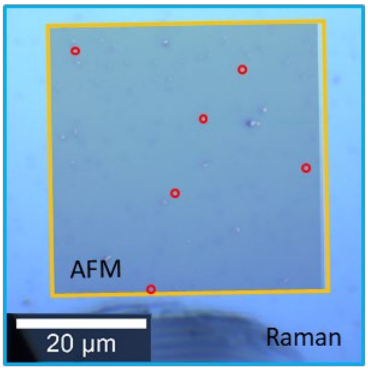

Figure S-2. Overview of AFM and Raman correlation image for time series measurement.

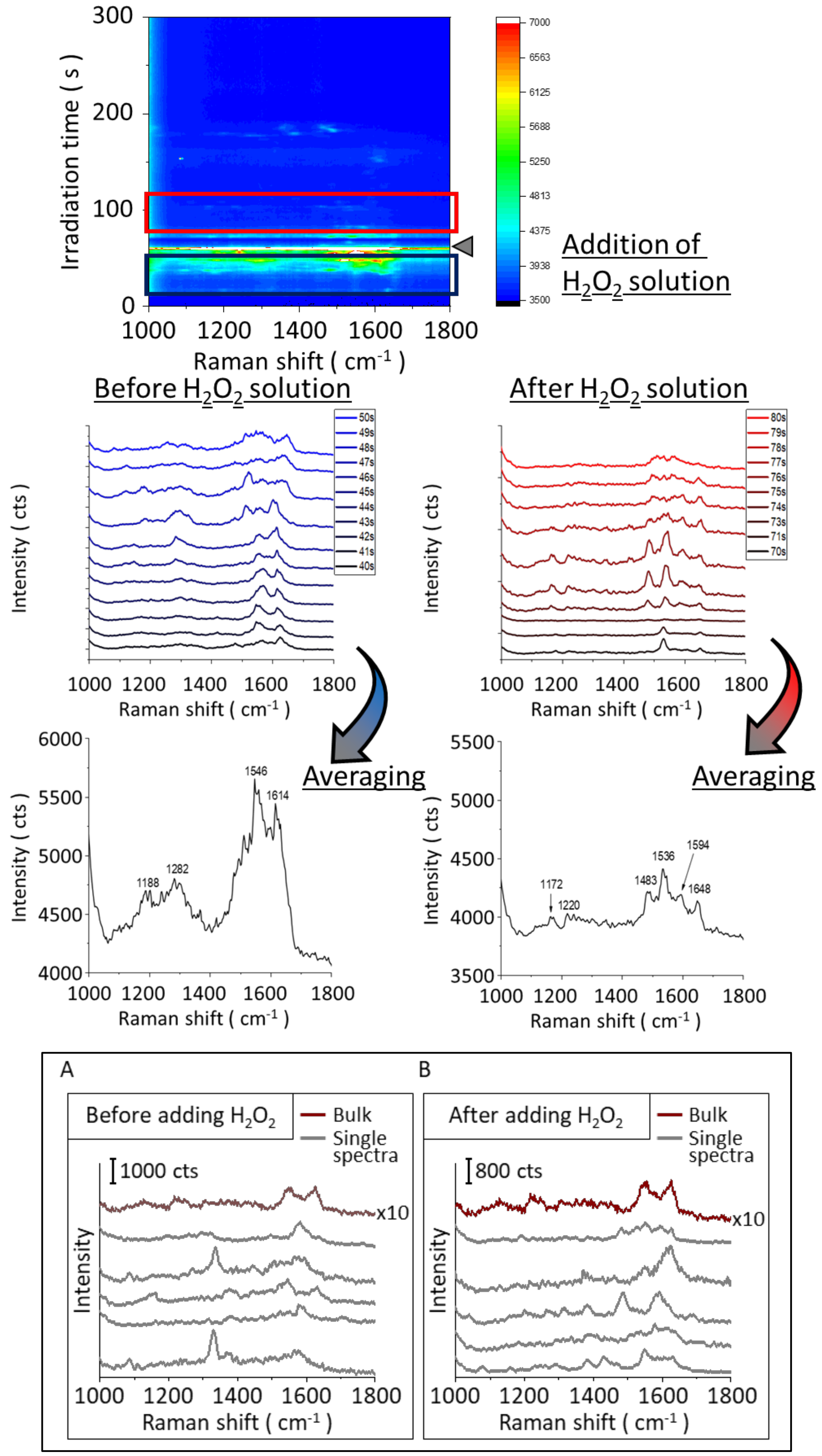

Figure S-3. Scheme of the data analysis: first, spectra are collected over time; after this, spectra with high signals are individually collected, and then the collected signal is averaged. Different averaged individual single-molecule SERS signals of HRP during the catalytic reaction are shown in the boxes

A and B (which represents Figure 3 of the main manuscript). Spectra were extracted before (left) and after (right) adding H_2O_2 solution.

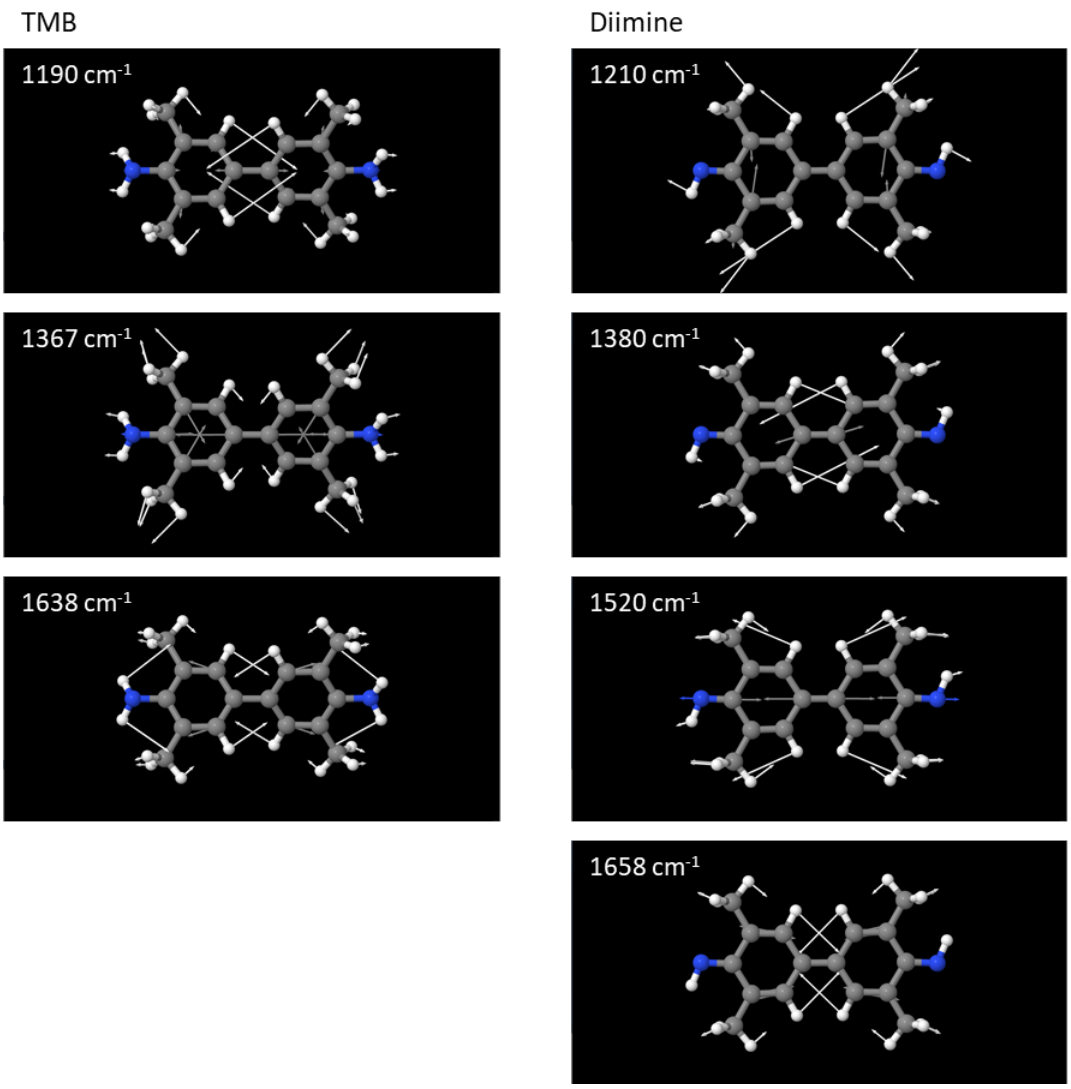

Figure S-4. Simulation of theoretical TMB and diimine vibrational modes. Vibrational modes that contribute to most pronounced bands in experimental SERS spectrum were shown.

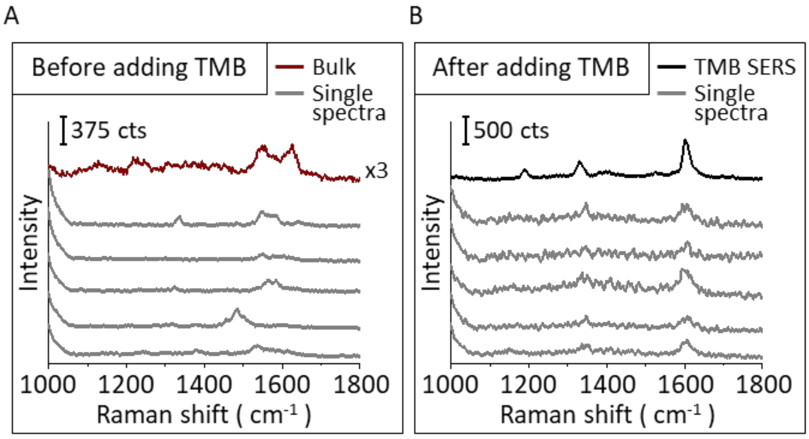

Figure S-5. Single-molecule SERS signals of HRP during the cyclic catalytic reaction. Spectra were extracted before (left) and after (right) adding TMB solution.

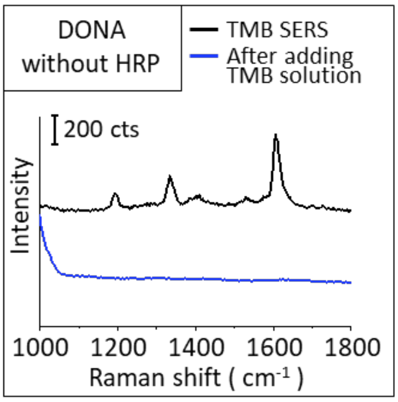

Figure S-6. Control experiment to investigate the interaction of AuNPs with TMB. Spectra were extracted after adding TMB solution to non-HRP-functionalized DONAs (blue), compared with TMB SERS spectrum.

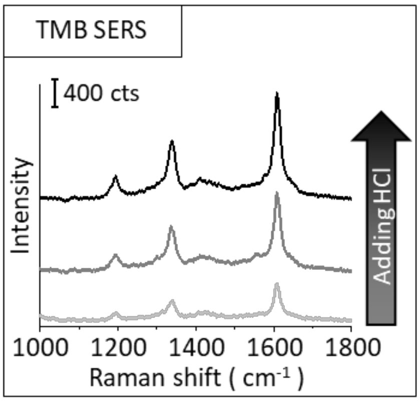

Figure S-7. pH dependency of TMB SERS signal. TMB was mixed with AuNPs and SERS signal was detected in liquid. HCl was added during the time series measurement and spectra were extracted from each adding HCl.

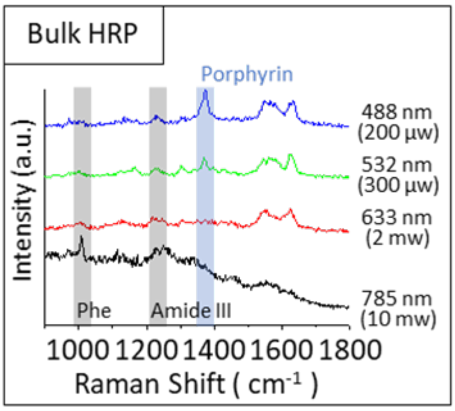

Figure S-8. Normal Raman spectra of bulk HRP at different excitation wavelength.

Table S-2. DNA sequences of staple strands which were utilized for the functionalization of HRP.

Name	Sequence (5' to 3')
S161	AAAGCACTAAATCGGAACCCTTGAATCGGCCAACGCGCGGGGTT-SH

Activity test of modified HRP

The assays were performed by checking the color change of TMB when oxidized by HRP. We mixed a TMB solution (0.5 mM) with the modified HRP, and incubated it for 5 min. If the enzyme is active, a blue color should appear. Figure S-9 shows the result for the HRP modified with DNA with different concentrations of HRP in solution.

We can see that even at the 3 nM concentration of HRP, we could observe a faint blue color, showing that the enzyme is active. This HRP concentration experiment determined the minimum concentration of NF needed to observe a color change. The second experiment was done with the DNA origami nanofork: first containing the nanofork without the SH-strand in the bridge and the other situation where the SH is present. Without the SH, it is expected that the HRP will not bind to the nanofork and will be washed away during the purification steps. Therefore, no color is expected. From the photography in Figure S-9, we can see the blue color in the solution for the sample where HRP should be present, while for the sample without the SH, the solution is still transparent, showing that there is no or not detectable non-specific binding of HRP to the nanofork and that the bound HRP is still active during our experiments.

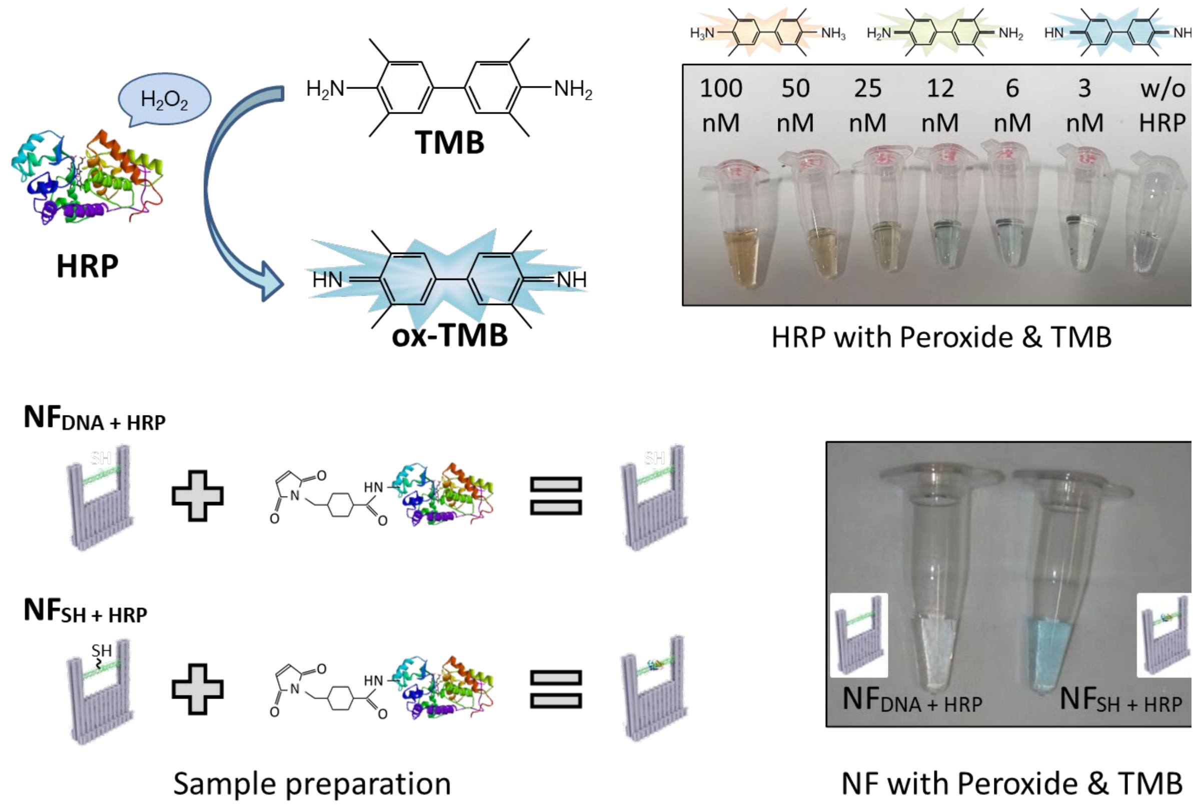

Figure S-9. Activity test of modified HRP.

References

(1) Rygula, A.; Majzner, K.; Marzec, K. M.; Kaczor, A.; Pilarczyk, M.; Baranska, M. Raman spectroscopy of proteins: a review. *J. Raman Spectrosc.* **2013**, *44* (8), 1061–1076. DOI: 10.1002/jrs.4335.

(2) Silin, V. I.; Talaikyte, Z.; Kulys, J. Surface-enhanced resonance Raman spectroscopy of microperoxidases and horseradish peroxidase adsorbed on silver hydrosol. *Vibrational Spectroscopy* **1993**, *5* (3), 345–351. DOI: 10.1016/0924-2031(93)87010-Q.

(3) Rakshit, G.; Spiro, T. G. Resonance Raman spectra of horseradish peroxidase: evidence for anomalous heme structure. *Biochemistry* **1974**, *13* (26), 5317–5323. DOI: 10.1021/bi00723a010.

(4) Rakhit, G.; Spiro, T. G.; Uyeda, M. Resonance Raman evidence for Fe (IV) in compound II of horseradish peroxidase. *Biochemical and biophysical research communications* **1976**, *71* (3), 803–808. DOI: 10.1016/0006-291X(76)90902-5.
